# Supplementary figures and images for: Quantitative mapping of mRNA 3’ ends in Pseudomonas aeruginosa reveals a pervasive role for premature 3’ end formation in response to azithromycin
Source: PLoS Genet. 2021 Jul 12;17(7):e1009634. doi: 10.1371/journal.pgen.1009634 (PMC8297930; doi:10.1371/journal.pgen.1009634)

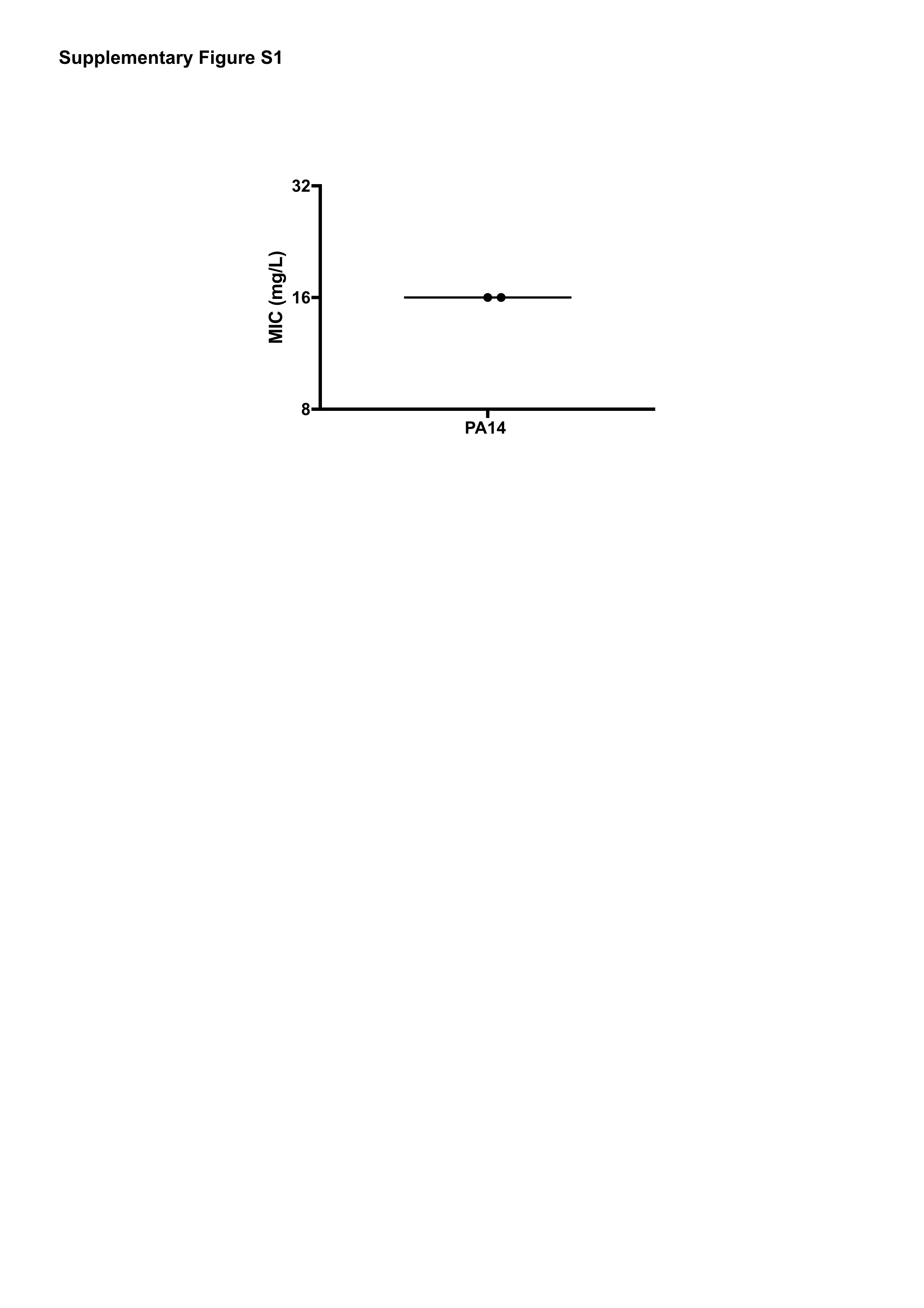

Supplement: S1 Fig — Three replicate assays were performed. Briefly, twofold microdilutions of Azithromycin dihydrate (Fisher J66740) in Mueller Hinton Broth were inoculated with 5x10^5^ CFU/mL PA14 in a 96 well plate. After 16–20 hours of incubation at 37°C, MIC was determined to be the lowest concentration of antibiotic at which no visible growth was observed. (TIFF) [file pgen.1009634.s001.tiff]

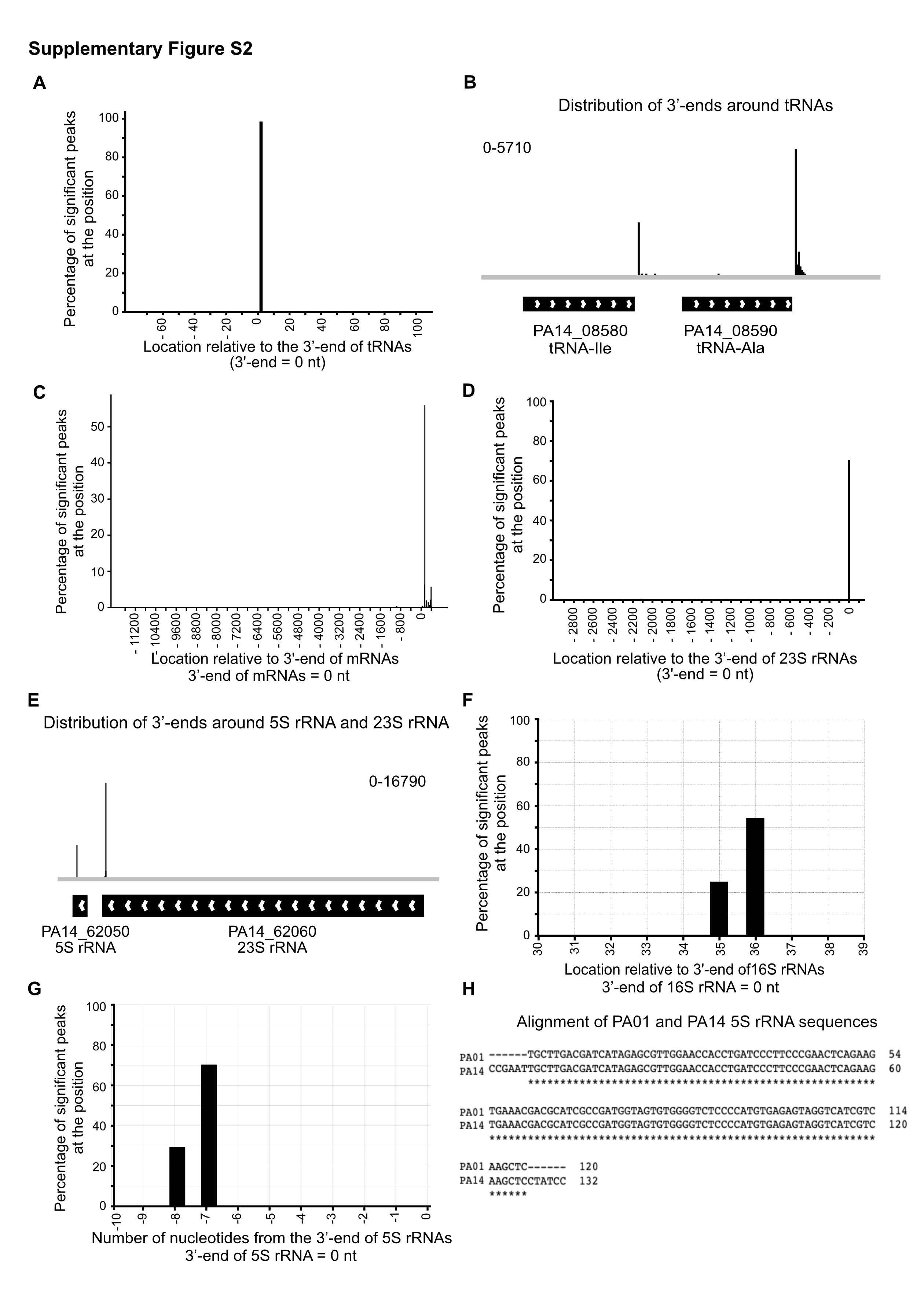

Supplement: S2 Fig — (A). Location of 3pMap reads with respect to the 3’ end of tRNAs (RNA end = 0 nucleotide). 98.78% of the significant peaks within or downstream of tRNAs are located within 3 nucleotides from the annotated RNA ends. (B) Genome browser view of distribution of 3’ ends around two tRNA genes. Number of 3’ end reads corresponding to each sample are denoted on the top of the sample tracks. (C) Location of 3pMap reads with respect to the 3’ end of CDSs. RNA 3’ end = 0 nucleotide. 88.4% of significant peaks associated with mRNAs were located in intergenic regions, with <12% located within coding sequences. (D) Location of 3pMap reads with respect to the 3’ end of 23S ribosomal RNAs. 99.8% of the significant peaks within or downstream of 23SrRNA genes are located within 3 nucleotides of the annotated RNA ends. (E) Genome browser view of the distribution of 3’ ends around representative 23S rRNA and 5S rRNA genes. (F) Extended 16S rRNAs in Pseudomonas aeruginosa. 80.54% of the detected 16S rRNAs (<10,000 reads in three replicates) had RNA 3’ ends in +35 and +36 nucleotides downstream of their annotated RNA 3’ ends. A recent study reported extended anti-Shine Dalgarno motifs in 16S rRNA in other bacteria, although they failed to identify such a motif in P. aeruginosa [132]. The major peak +35/36 downstream of 16S rRNA could represent extended anti-Shine Dalgarno motifs in P. aeruginosa 16S rRNA, capable of providing additional specificity or transcript preference for Shine Dalgarno motifs on mRNA, or unprocessed pre-16S rRNAs [132, 133]. (G) 99.9% of RNA 3’ end significant peaks associated with 5S rRNAs were located -8 or -7 nucleotides upstream of their annotated RNA ends. (H) Alignment of 5S rRNA sequences from PA01 and PA14 genome annotations using Clustal Omega (www.ebi.ac.uk). The prokaryotic 5S rRNA sequence is well conserved and 120 nucleotides long, including in the PAO1 strain (Pseudomonas genome database). We aligned the 5S rRNA sequences from PAO1 and our PA14 using [file pgen.1009634.s002.tiff]

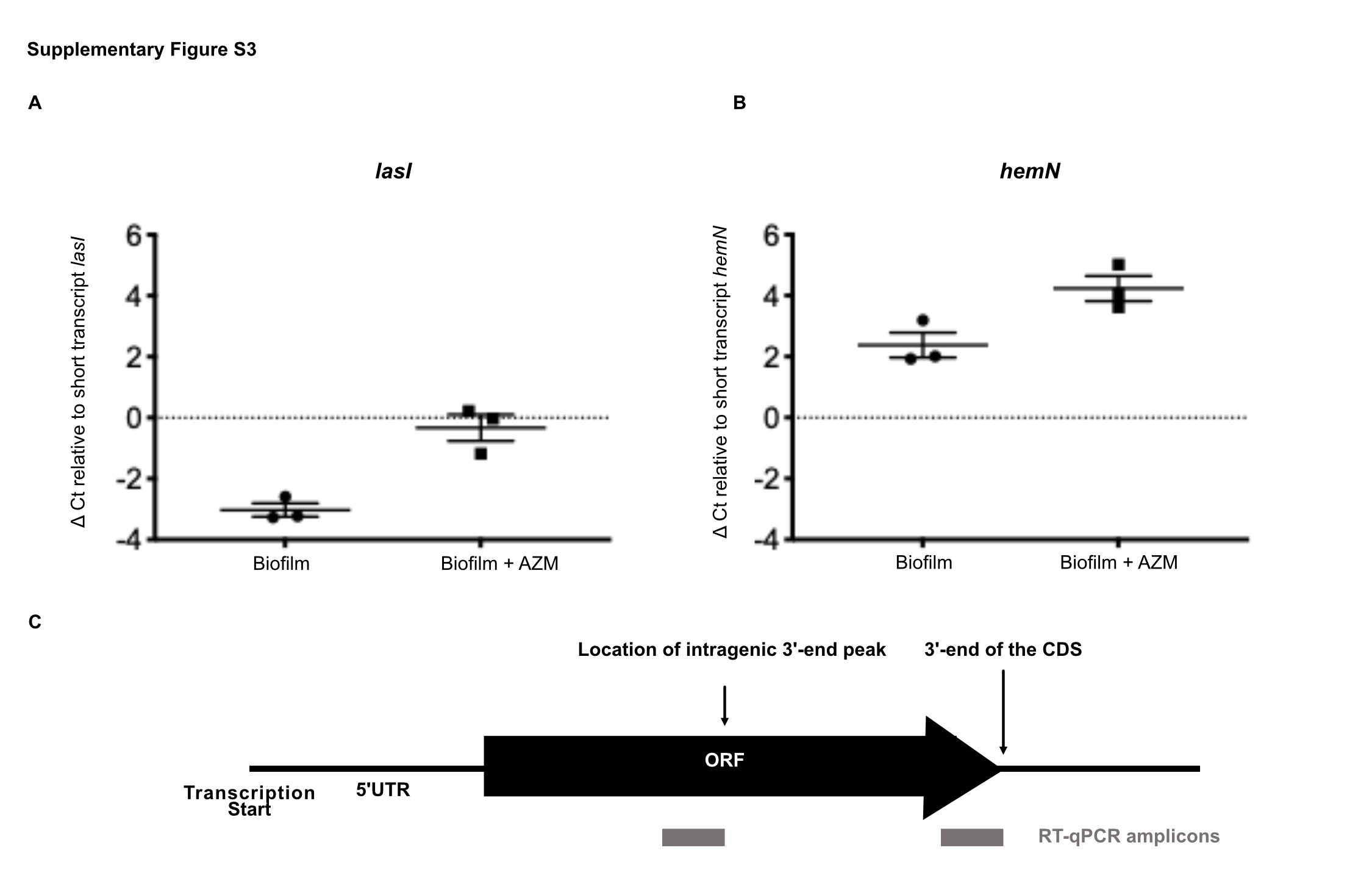

Supplement: S3 Fig — Total RNA was phenol-chloroform extracted from biofilm samples and treated with 1 unit of RQ1 RNase-Free DNase (Promega M6101) according to manufacturer’s protocol to remove any residual genomic DNA. DNase-treated RNA was reverse transcribed using a 2 μM pooled mixture of reverse primers for the target genes of interest (S14 Table) and Superscript IV Reverse Transcriptase (Thermofisher Scientific 18090010). The reverse transcription reaction for intragenic and full-length transcripts were performed for 20 minutes at 55°C. cDNA was amplified with the corresponding primer pairs (S14 Table) for targets of interest and Sybr Green PowerUp Master Mix (Thermofisher Scientific A25741) according to the manufacturer’s protocol on Applied Biosystems StepOne Plus Real-Time PCR system. For each condition, three biological replicates were tested, with three technical replicates for each. (TIFF) [file pgen.1009634.s003.tiff]

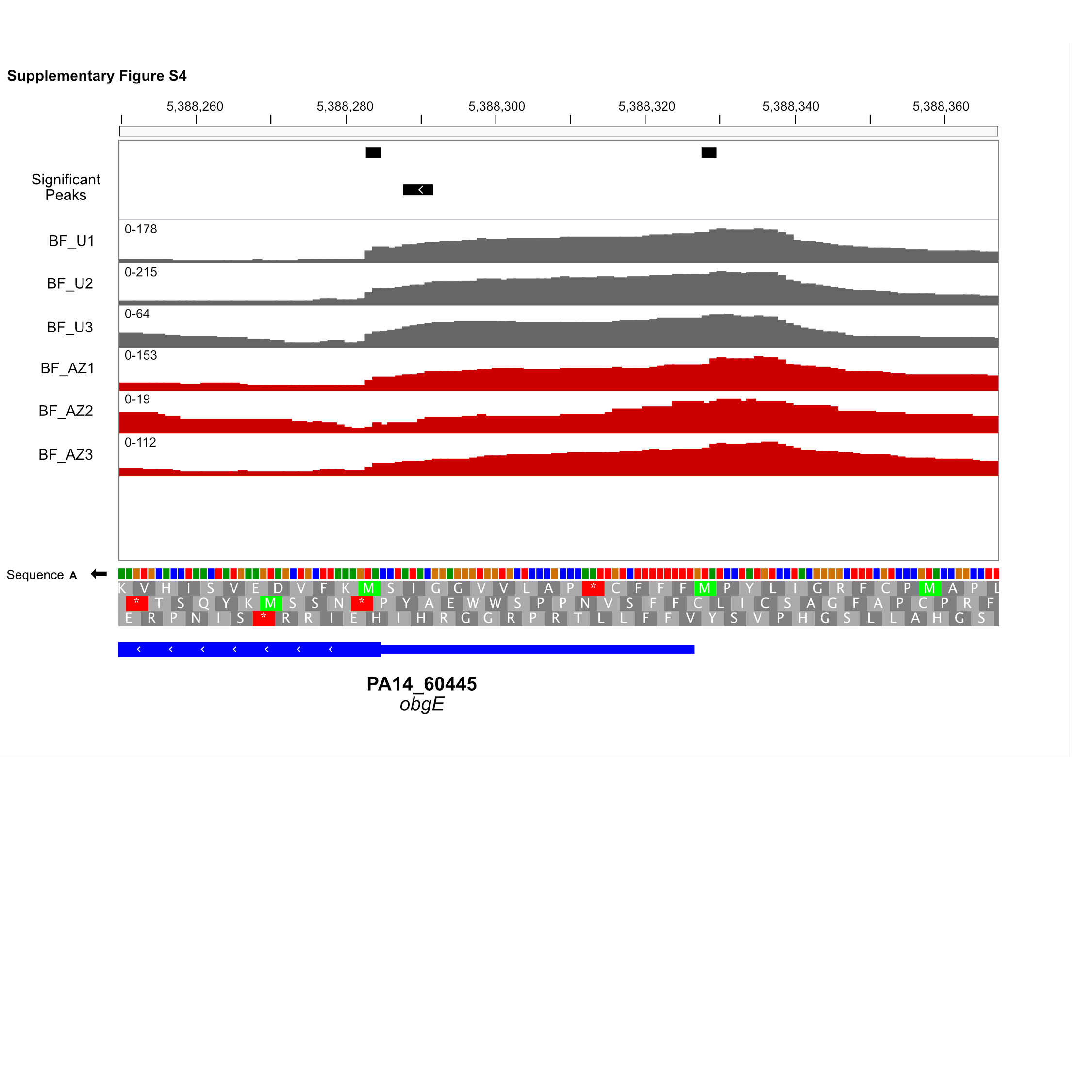

Supplement: S4 Fig — Tracks for all three replicates of biofilm treated (AZM) and untreated (U) samples are shown. The numbers on the scale depict original, non-normalized read counts. Both AUG methionines of the potential uORF, though outside the annotated transcript leader from Wurtzel et al., are covered by transcribed RNA in vivo. Note the strong accumulation of 3’ ends at the obgE start codon, which appears as a “cliff” before coverage drops (compare to Fig 3). (TIFF) [file pgen.1009634.s004.tiff]

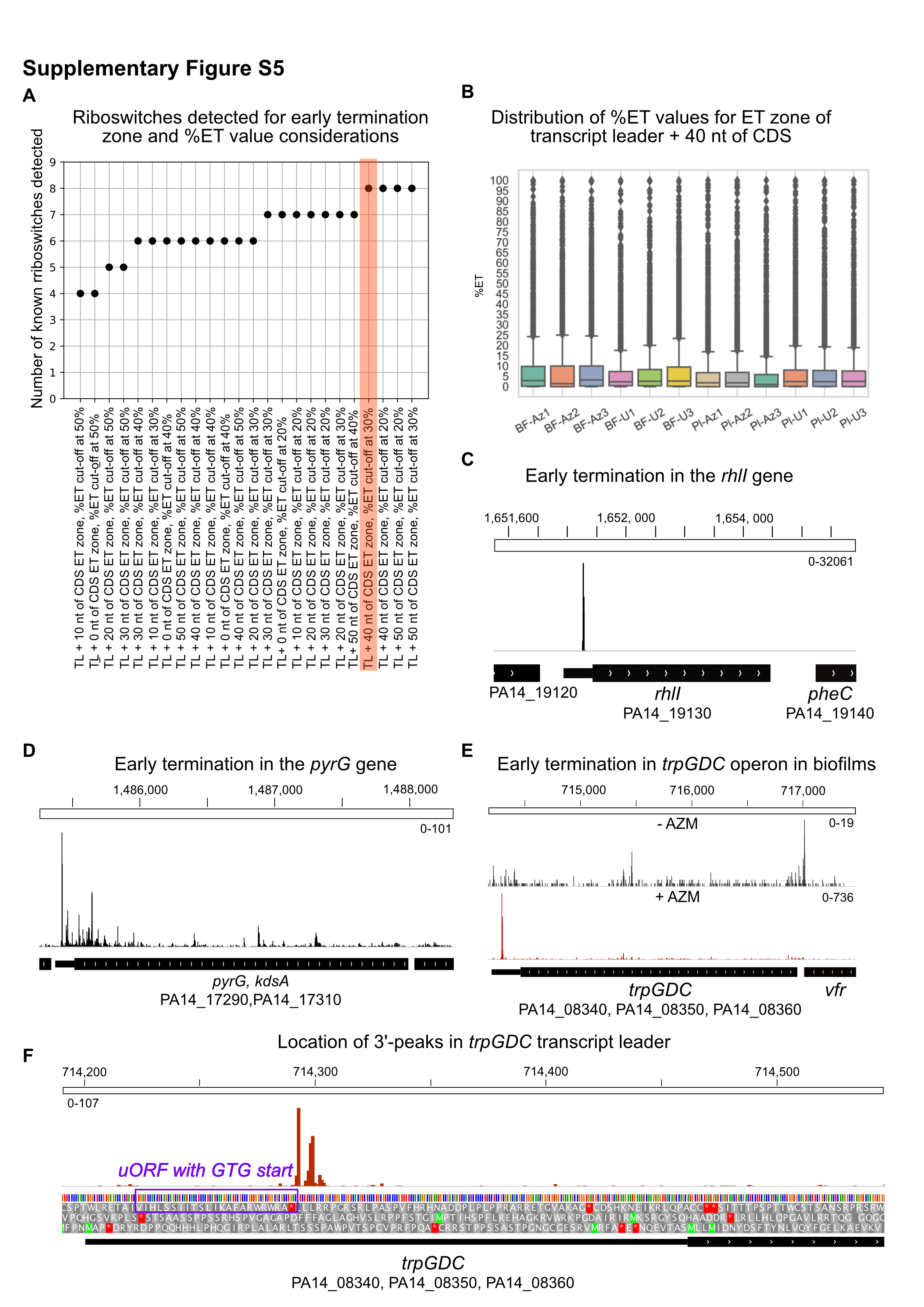

Supplement: S5 Fig — (A) Determining the early termination zone. Number of known riboswitches reported on Rfam detected for each ET zone and %ET cut off considerations. The lowest ET zone consideration of transcript leader + 40 nt in CDS and the highest cut-off value of 30% detected 8/11 riboswitches reported on Rfam, and was therefore used for predicting putative riboregulators. (B) Box Whisker plot showing the distribution of %ET values in samples using ET zone transcript leader + 40 nt of CDS. (C) Early transcription termination in the rhlI gene detected by 3pMap [133]. Genome browser images show strand-specific tracks for the strand same as the gene of interest and in untreated planktonic samples unless specified. (D) Potential regulation of the pyRG gene by in P. aeruginosa by 3’ end formation in its transcript leader. The pyrG expression is controlled by transcription attenuation in B. subtilis [134]. Genome browser image shows pyrG, kdsA operon. (E) Premature 3’ end formation in trpGDC operon in P. aeruginosa biofilms. %ET values in planktonic (untreated and +AZM) and untreated biofilm samples were below the cut-off value. However, in AZM-treated biofilms (+AZM), regulation by premature 3’ end formation was predicted. The gene is predicted to be regulated by transcription attenuation in B. subtilis [135]. (F) A closer view of the transcript leader and early termination peak in trpGDC operon. The 3’ end peaks are located adjacent to a potential uORF with GTG start codon. (TIFF) [file pgen.1009634.s005.tiff]
