# Supplementary material for: Quantitative mapping of mRNA 3’ ends in Pseudomonas aeruginosa reveals a pervasive role for premature 3’ end formation in response to azithromycin
Source: PLoS Genet. 2021 Jul 12;17(7):e1009634. doi: 10.1371/journal.pgen.1009634 (PMC8297930; doi:10.1371/journal.pgen.1009634)
Supplement: Attachment — Submitted filename: Response-to-Reviewers.docx [file pgen.1009634.s021.docx]

Reviewer's Responses to Questions

**Comments to the Authors:
Please note here if the review is uploaded as an attachment.**

Reviewer #1: In the manuscript entitled “Quantitative mapping of mRNA 3’ ends in Pseudomonas aeruginosa reveals putative riboregulators and pervasive role for transcription termination in response to azithromycin”, the authors present collection and analysis of a 3’-end RNAseq dataset for planktonic and biofilm P. aeruginosa in the absence and presence of sub-MIC azithromycin (a translation inhibitor). The strengths of the work are a rational analysis method for identifying significant peaks from what is typically noisy data generated by 3’-end sequencing, and a number of interesting findings. These include: a DE analysis of biofilm vs. planktonic cells + and – azithromycin, analysis of how early termination may impact this response, and some observations of key genes that appear to be impacted by early termination. There are quite a few nice little stories presented here, several of which are quite interesting results.

However, the work lacks a single clear story or compelling conclusion. The titular finding is not surprising given what is known about translation inhibition in gram-negative bacteria generally. Nothing has been followed up experimentally beyond the initial data collection, not even qPCR to validate some of the more interesting results (such as the potential tRNA charging differences). The authors could have more impact by focusing on one specific result and demonstrating its biological relevance rather than listing a scattershot of things that popped up from their analysis.

My major issues with the manuscript are below, and many of these are likely addressable with clarified writing.

We thank the reviewer for their careful consideration of our manuscript. We believe that the scope of the manuscript, encompassing multiple aspects of the *P. aeruginosa* response to AZM, is appropriate for PLoS Genetics given the genomic nature of the study. However, we have revised the manuscript for general clarity and to more clearly focus the results on our investigation of the transcriptional response to AZM in biofilms and planktonic culture. While we agree that the occurrence of early transcription termination in response to AZM could be predicted in a general sense, the extent to which it influences the broader transcriptional response is surprising and novel. Prior work in *P. aeruginosa* has not considered the role of early transcription termination. In particular, the *lasI* gene was previously shown to have decreased expression in response to AZM by qPCR, and this was proposed to be a result of transcriptional downregulation. Our results show that *lasI* is actually transcriptionally up-regulated, but subject to massive early termination. Our revision also includes a new analysis of rho-dependent vs. intrinsic termination, qPCR validation of changes in the relative frequency of internal and full-length transcript formation for two genes, and extensive text revisions to clarify our work and provide a more balanced consideration of alternative interpretations. We believe the revised manuscript is much improved through these changes and thank the reviewer for their constructive comments. Responses to individual concerns are detailed below.

1. 3pMAP – authors claim this is an improved approach in the abstract. While it has fewer steps, and requires less material no data are presented to directly demonstrate that it is improved over TERMseq. In particular, since TERMseq is typically accompanied by traditional full-transcript RNAseq, one of their references (Ma et al. BMC Genomics 2019) would argue that the reverse is true. I think it probably does the job, but whether it is a second ligation step, or a PCR step with a random primer may in fact change findings, and this is not addressed by the claim.

As we did not benchmark 3pMap against TERM-seq or other 3’ end mapping approaches, we agree with the reviewer that it is appropriate to not call it an improved method. We replaced the wording of “an improved” with “a fast, cost-effective” method to more accurately describe the benefits of 3pMap in the abstract.

2. The authors use the known riboswitches in P. aeruginosa to calibrate the threshold for what constitutes a significant percentage of early termination events. Of the riboswitches identified and used, how many are expected to act through transcription termination vs. other mechanisms such as translation inhibition (which could indirectly impact either rho-dependent termination or mRNA decay)?

The riboswitches used to calibrate the percentage cut-off values were obtained from Rfam and act via translation inhibition in gram-negative bacteria (5 cobalamin, 1 TPP, 1 FMN and 1 SAH riboswitches), premature transcription termination (1 fluoride), transcription/translation regulation (2 yybP-ykoY manganese riboswitches). The same riboswitches were identified by Riboswitch Scanner as well, with no new predictions. The positive candidates detected by the cut-off included 5 cobalamin riboswitches, 1 TPP riboswitch, 1 fluoride riboswitch, and 1 manganese riboswitch (Supplementary Table S11).

Do the authors expect these riboswitches to be in the “terminated” form under the conditions which they collected their data?

Even in rich media conditions, some cells may be deficient for particular small molecules and metabolites, while others have more than enough. Thus, we expect riboswitches should always have some fraction that are in the terminated form (be it ever so small), and our ability to detect them depends on the sequencing depth and thresholds used to classify early termination genes. To be conservative, we used a 30% threshold for early termination, as this was the lowest level required to identify known riboswitches (Figure S5; new version now legible).

3. I am a little confused how operons and multi-cistronic genes would be impacted by the analysis method if indeed only 3’ mapping data was used for analysis. It seems that 1) all 3’ ends are captured. 2) 3’ ends are associated with a gene by mapping and can be classified as either “pre-mature” or not. What about genes which are expressed, but do not have a termination event within or immediately following them? How is expression of these genes quantified? Perhaps it doesn’t matter if AZM doesn’t impact them? The Ma et al. referenced above suggests that 3’-mapping does do better with finding shorter rather than longer transcripts, how does this square with the average transcript length expected in P. aeruginosa?

The reviewer raises an interesting point. Ma et al. compared 3’ end mapping to traditional full-transcript RNA-seq using mouse liver mRNA. They found 3’ end mapping was more effective at detecting shorter transcripts (<2,500 nucleotides in mouse), and equally effective at detecting long transcripts. Even considering multi-cistronic operons, as defined by Wurtzel et al. (Table S2), the vast majority of *P. aeruginosa* transcription units are less than 2,500 nucleotides (see histogram below). Thus, we expect 3’ end mapping will be at least as sensitive as traditional RNA-seq for detection of RNA transcripts in *P. aeruginosa*, and may be more sensitive based on Ma et al.

4. On the tRNA charging quantification, it seems to be it would be difficult to know just from a reduction in accessible 3’-OH, whether this is less tRNA, or inaccessible 3’-OH due to charging. Can previous data or qPCR be assessed to control for this? This actually strikes me as one of the most interesting tidbits to come out of this work, but without this control it isn’t a convincing finding.

We thank the reviewer for raising this issue. We observed an increase in free 3’-OH ends of several tRNA in response to AZM treatment. This increase could indeed result from decreases in tRNA charging or from increases in tRNA transcription. Unfortunately, quantifying total vs. charged tRNA by qPCR is challenging due to reverse transcriptase inhibition by common chemical modifications in tRNA. While northern blot analysis with and without chemical deacylation could help differentiate these possibilities, we were unable to carry out such experiments due to reduced wet-lab operational capacity during the pandemic. Thus, we were not able to determine whether tRNA level changes result from changes in aminoacylation or transcription levels. To address this, we have revised the manuscript to clearly describe both possibilities and their expected impact on translation, including a more conservative interpretation of this result (lines 331-333, 509-529) .

5. The first mention of rho is on page 17 (there is a reference to rho in figure 5 as well). Discussion of whether these new termination sites are likely to be rho-dependent or rho-independent is very important. Inhibition of translation could easily cause increased rho-dependent termination in gram-negatives such as P. aeruginosa (this is likely the predominant cause of early termination observed!). Conflating rho-dependent and independent termination (as the authors do since they do not explicitly mention rho until the very end of the manuscript) is misleading.

We agree with the reviewer that the first submission did not adequately discuss the relative roles of rho-dependent and independent termination. In the revised manuscript, we added language describing these termination modalities to the introduction and performed a new computational analysis to predict Rho-independent terminators using ARNOLD to the first subsection of the results. We find that roughly one fifth of reads in significant 3’ end peaks correspond to predicted rho-independent terminators, suggesting most termination is rho-dependent. This is consistent with your suggestion that the increased 3’ end formation we see is due to translation inhibition and rho-dependent termination.

6. Along the same lines, PASIFIC analysis assumes a rho-independent termination event (and the formation of a rho-independent transcription terminator). The rho-independent terminators shown in figure 2C are not very convincing as terminators, the expected polyU is weak in both terminator examples shown. If these are their best examples, what do the low confidence ones look like?

The reviewer is correct in noting that PASIFIC predicts rho-independent riboregulators, classically characterized by stable stem loop structures followed by U-rich stretches. The *lasR* and *gyrA* examples were chosen to use in Figure 2C based on their previously described functions in QS and drug-resistance, and do not represent textbook examples of rho-independent terminators. To further examine this, we updated Table 1 in the revision to show the location of ARNOLD predicted rho-independent terminators on PASIFIC predicted riboregulators. 12.5% of PASIFIC predicted riboregulators have rho-independent terminators predicted by ARNOLD. In the revised manuscript, we have moved the section on predicted riboregulators to the end of the results section, and added language to reiterate these are predicted, putative regulatory elements to both the results and discussion. We believe these changes help focus the manuscript on the transcriptional response to AZM and send a clear message that these are predicted elements whose further testing could be the subject of future studies.

7. Furthermore, PASIFIC was trained on microbiome organisms (and tested only on gram-positive organisms in the published work), these may have very different terminator profiles than *P. aeruginosa*. Doing this type of analysis well is difficult, but the authors might find some approaches in this review that would be helpful for identifying alternating structures that do not depend on formation of intrinsic terminators (Fukunaga and Hamada 2018 <https://pubmed.ncbi.nlm.nih.gov/30689706/>)

We thank the reviewer for bringing to light the challenges of riboswitch prediction using computational approaches. The review article they suggest listed two riboswitch prediction programs in addition to PASIFIC - Riboswitch Scanner, and Riboswitch Finder. These approaches largely use sequence homology to find candidates that are similar in sequence to known riboswitches. In our hands these gave results identical to those of searching Rfam (Scanner) or no results at all (Finder). The latter approach may have given no results because it is 17 years old and hasn’t been updated. In the revised manuscript we have extensively edited the section on riboswitches and attenuators to emphasize that these are predicted elements that would require validation in future studies outside the scope of our current work.

Minor issues:

1. rplJ is known to be regulated by an RNA regulator that is homologous to the one characterized in E. coli (Fu et al. 2013 <https://www.ncbi.nlm.nih.gov/pmc/articles/PMC3616713/>).

We thank the reviewer for pointing this out and have added the reference to the revised manuscript.

2. trpGCD is known to be regulated by attenuation in Pseudomonas putida, and a homologous leader is present in P. aeruginosa. (Olekhnovich and Gussin 2001 <https://www.ncbi.nlm.nih.gov/pmc/articles/PMC95228/>). I would double check with a deep literature dive other examples you cite as regulated in other organisms but not in P. aeruginosa, especially if E. coli regulation is known.

We thank the reviewer for noting this missing reference, and we have added it to the manuscript. We were unable to find additional examples of our new riboregulator candidates that were previously predicted in other species.

3. The closest relative to P. aeruginosa that contains a T-box is a delta-proteobacteria (thought to be acquired from horizontal transfer)(Kreuzer and Henkin, 2018 <https://www.ncbi.nlm.nih.gov/pmc/articles/PMC6329474/>). I would not invoke them in the context of P. aeruginosa repeatedly.

We agree with the reviewer and have removed all language discussing T-boxes from the revised manuscript.

4. Much of the text in Figure S3 is illegible in my review copy).

We apologize for the poor rendering quality of the figure in the initial submission and have updated a clear version in the revised manuscript (now Figure S5).

Reviewer #2: In their manuscript, “Quantitative mapping of mRNA 3’ ends in Pseudomonas aeruginosa reveals putative riboregulators and a pervasive role for transcription termination in response to azithromycin”, Konikkat et al. use a modified RNA sequencing approach to describe the effects of subinhibitory azithromycin treatment on transcript 3’ ends. This is an interesting data set that has value for a broad audience. Layers of regulation affecting transcription attenuation, translation efficiency, and transcript stability have mostly been investigated in E. coli, leaving many open questions about the roles of these mechanisms in other bacteria, including important pathogens, and the experiments presented here contribute to addressing that knowledge gap. However, in places the conclusions seem substantially overstated relative to the data supporting them, and there are some questions surrounding the data analysis that could likely be resolved by providing more detail in the materials and methods and/or addressing possible errors. Major concerns are detailed below.

The authors are grateful for the reviewer’s kind appraisal of the value of our study. We have added new qPCR validation data and additional computational analyses to address the concerns they raised, and have substantially revised the manuscript to avoid overstating conclusions and clarify the data analysis issues raised. Many of the reviewer’s concerns were, understandably, lengthy. To ensure we addressed each aspect of these concerns, we attempted to break down some of the numbered sections into individual points in our response below. We believe the revised manuscript is much improved as a result of addressing the issues raised.

Data analysis:
The data analysis in a non-standard NGS experiment is non-trivial, and while the details provided in the materials and methods are appreciated, in places even more detail should be provided. Additionally, this data set has the potential to serve as a useful resource to many other researchers, since it contains genome-scale information about 3’ transcript ends. To fulfil this role to the extent possible, it would be helpful if the supplemental tables were described better in places as noted below. Finally, it would be useful to include a supplemental table describing the positions of significant 3’ end peaks for each transcript for which they could be detected – this information about putative transcription termination sites would help many researchers designing genome modifications or expression constructs to make accurate choices about transcript boundaries. Depositing the raw data in an NCBI database is appropriate and appreciated, however I might suggest that the GEO database would be the most appropriate and helpful place to deposit these data. This database allows tables of processed data to be associated with the raw data, includes standardized and detailed information about the data analysis, is manually curated, and allows private links to be provided for reviewers prior to publication of the data.

We agree with the reviewer that the 3’ ends we identified will be a rich resource for future research. As such, we have added a new supplemental table listing the significant 3’ end peaks associated with each gene (Table S3). These and other supplementary tables will be available as open access resources if, as we hope, the manuscript is published at PLoS Genetics. In addition, we have deposited supplemental files to NCBI GEO, including strand-specific bedGraph files of 3’ ends for each of the 12 samples, a bed file listing all significant 3’ end peaks, and the new Table S3 described above. Finally, we also included our custom scripts as a supplemental file.

1. Separated into individual points for responses.

a. The authors cite Wurtzel et al. (PloS Pathogens, 2012) as the source for their transcription start site definitions, but claim that this study was performed in the PAO1 strain and describe a procedure for “transferring” the annotations to PA14. This is definitely incorrect – the Wurtzel et al. study was performed in a PA14 strain according to the published description, and it is clear from their supplemental files that the genome they used is a PA14-like genome. It is possible that there are minor differences between the genome sequence used by Wurtzel et al. and the genome sequence used in these studies. Supplementary table 7 does seem to reflect some differences in the exact nucleotide positions of transcript starts and stops relative to the Wurtzel et al. supplementary tables. This could be in part due to confusion related to strain names – the NCBI reference sequence ID listed in supplementary table 7 is for the commonly used strain whose complete name is UCBPP-PA14, and there is at least one other published isolate genome called “PA14” that is distinct. Both Wurtzel et al. and the current manuscript use only “PA14” to describe their strains, so there is some ambiguity. If the strain used here was actually UCBPP-PA14, it would be helpful to state this clearly somewhere in the manuscript.

We thank the reviewer for bringing this to light. Indeed, we used the Cooper lab strain of UCBPP-PA14 which is nearly identical to the PA14 used by Wurtzel et al, with few differences in their genome sequences. We show an alignment of the two strains using MAUVE (Darling lab) below, which shows the high degree of identity (99.996% identical). There are 203 SNPs and 61 gaps, almost all of which are single nucleotide indels. We have updated the text to clarify this point.

b. Additionally, the references to Wurtzel et al. using PAO1 should be corrected, and the reason for the described procedure to transfer annotations should be clarified.

As the reviewer suggested, we have corrected the manuscript to list PA14 as the strain used in Wurtzel et al. We have also updated the text to explain that the Wurtzel PA14 strain and our strain are nearly identical. We used liftOver to transfer the Wurtzel annotations to the corresponding coordinates in our strain to account for the gaps in the strain alignment.

c. Finally, in the method for transferring the annotations, it is noted that if a 5’UTR was predicted to overlap the upstream ORF, the ORF boundary was used as the boundary of the downstream transcript. This seems unjustified. There are many examples in multiple organisms of promoters and transcription start sites falling within ORFs.

The reviewer is correct in pointing out that transcription start sites can fall within ORFs. We believe the wording used to explain the reasoning underlying our transfer procedure in the original manuscript may have been unclear. In overlapping cases, the downstream transcript leader was removed from the annotation to favor assigning 3’ ends as “normal” transcription termination sites to the upstream gene, as opposed to calling them riboregulatory termination sites. We felt this was a conservative compromise to limit false-positive predictions of premature transcription termination sites. This removal of Wurtzel transcript leaders occurred at 271 of the 3,794 transcription units in the Wurtzel annotation (7.1%).

2. All of the supplementary tables seem to use transcript definitions that acknowledge some polycistronic transcripts, as is appropriate. However, the method used for defining operons is unclear. As mentioned above, supplementary table 7 seems to be derived from Wurtzel et al., but for several genes, the operon definitions used in the other supplementary tables is different from that in table 7. The reasons for this should be clarified.

We apologize for any issues encountered in our initial supplemental tables. We have added language to the revised methods section to clarify how we obtained transcript models for genes and operons using the liftOver process from Wurtzel et al. We believe the supplementary tables in the revised submission are free of such issues.

3. Supplemental table 7 is described as “Bed file used for assigning 3'-end reads and peaks to transcript leaders, intragenic, and 3pUTR categories.” but does not seem to contain sufficient information for fulfilling this role – it has only a single transcript start and end position per transcription unit. The additional required information for defining these regions should be added.

We apologize for any confusion created here. Table S7 is not a standard bed file, because we joined all the ORF sequences of each polycistronic transcript together in one internal block. The table shows a single TSS and a single termination site for each gene based on the Wurtzel annotation after adjusting TSSs to avoid overlap with upstream transcription units. Because this comes from the Wurtzel annotation, which has single TSS and termination sites (end of last ORF) for each transcription unit, our adjusted file also has only a single TSS and termination site. In the revised submission, we include a new supplementary table 13, which has all significant 3’end peaks for each gene. We have also revised the description of Table S7 (New supplementary table S2) to clarify that it is not a bed file format.

4. Supplementary table 2 appears to be in a random order and has blank lines.

We have taken care to address this issue in the revised manuscript. Due to the addition of new supplementary tables and revisions of the manuscript this table is now Table S12

5. Supplementary table 6 could be better described. I assume it contains read counts for reads associated with significant peaks in the early termination zone and full length zone for each transcript and replicate, and that these values were used in the CMH statistical analysis.

a. However, these assumptions are inconsistent with other aspects of the described analysis.

i. The materials and methods report using a pseudocount of 1 for regions with no peak reads, but there are zeros recorded in the table.

The pseudocounts were used internally in R for statistical analysis, and were not reported in the supplementary table. The R code used is now included as a supplemental file.

ii.Furthermore, it seems that using a pseudocount of 1 could in some cases have a significant impact on the outcomes, because the actual counts leading to statistically significant results are quite low in some cases.

We thank the reviewer for bringing this important issue to our attention. For some genes, no intragenic termination sites were observed in the data for any replicate. In such cases, pseudocounts could indeed lead to apparent statistical significance, when, e.g., the pseudocount corrected ratios change from 1 / 1000 in untreated samples to 1/3000 in treated samples. To correct this potential problem, we restricted our analysis in the revised manuscript to transcript units that have no more than one “zero” value per treatment-triplet set (i.e. one zero in all three replicates of intragenic and / or one zero in all three replicates of full-length in both treated and control samples) (lines 221-224). This increased stringency reduced the analysis to 893 genes in planktonic culture and 861 genes in biofilms. Importantly, the general conclusions remained valid and were, in fact, strengthened by this reanalysis as 93% of genes downregulated in biofilms treated with AZM were found to have significant increases in premature 3’ end formation. We updated the revised text to clarify this and report the new results (lines 356-388).

iii. Also, the counts recorded here seem to reflect wildly different percentages of early termination for some genes compared with the values reported in supplementary table 2. I understand from the materials and methods text that table 6 assessed reads associated with significant peaks while table 2 used all reads in the two regions, but both tables report assessments of the prevalence of early termination, and the reasons why they give very different numbers for some genes should be addressed.

The difference is that the original Table S2 only included the transcript leader region up to 40 nt into the CDS, while Table S6 included the whole transcript leader and CDS. Table S2 was calculated to search for riboregulators, which traditionally are in the 5’ transcript leader or 5’ end of the gene. This is described in the methods section of the revised manuscript (lines 217-246).

b. Finally, table 6 does not appear to include data for the planktonic condition, but statistical analysis of early termination in this condition is described in the text.

We apologize for the confusion in the first submission. Data for the planktonic condition were present, but separated by several blank columns and shifted to the right of the table. We have addressed this in the resubmission by separating biofilm and planktonic results into two separate supplemental tables (S5 and S6)

6. Several references to custom scripts appear in the materials and methods. Using custom scripts is often required and acceptable, but what these scripts do should be better described.

We agree with the reviewer and have updated the methods extensively to better describe the functions of custom scripts. We have also included all custom scripts as supplementary files.

a. For example, It is not clear if or how read counts were normalized to library depth for the DESeq2 analysis.

We apologize for not explicitly stating this in the original manuscript. DESeq2 was run using standard parameters. We did not normalize the input data. We have added language to clarify this in the methods section of the resubmission.

b. It is also not clear if the definition of “significant” peaks strongly penalizes broader peaks with reads spread over several nucleotide positions. Such broader peaks are expected from some mechanisms for transcription termination, and could be missed if the analysis method strongly favours 3’ ends that are specific to a single nucleotide position.

We agree with the reviewer that this is an important consideration. Our computational analysis searches windows for nucleotides that have an unusual, statistically significant enrichment of 3’ ends using a Poisson test. We then merged significant sites. In practice this could preclude the identification of very broad peaks. To evaluate the effects of our peak calling algorithm, we examined the percentage of all aligned 3’ end reads that were assigned to significant peaks (both from genes and non-genic regions)**.** With the exception of one AZM-treated sample (Planktonic Replicate 3), over 75% of reads were used in all samples, with a median usage of 83.33%. Furthermore, manual evaluation of browser tracks did not reveal broader “peaky” areas that were overlooked by our automated analysis. Thus, we believe our peak calling algorithm accurately identifies significant 3’ end termination loci while removing noisy 3’ end loci.

| Sample | Total no. of 3'-ends in peaks | Total no. 3'-ends in reads | Percentage of 3'-ends that are associated with peaks. Formula = (peaks*100)/reads |
| --- | --- | --- | --- |
| BF_Az1 | 8265159 | 9076731 | 91.06 |
| BF_Az2 | 977610 | 1168546 | 83.66 |
| BF_Az3 | 4873423 | 5634885 | 86.49 |
| BF_U1 | 6337445 | 7560793 | 83.82 |
| BF_U2 | 5543060 | 6716193 | 82.53 |
| BF_U3 | 5225029 | 6076788 | 85.98 |
| Pl_Az1 | 4303318 | 5401682 | 79.67 |
| Pl_Az2 | 3788253 | 4830991 | 78.42 |
| Pl_Az3 | 1454397 | 2981556 | 48.78 |
| Pl_U1 | 6272773 | 7557506 | 83 |
| Pl_U2 | 3826949 | 4970141 | 77 |
| Pl_U3 | 5072788 | 5982646 | 84.79 |

Other issues
1. Results relating to rRNA transcripts cannot generally be assumed to be valid when samples have been treated to deplete rRNA. The depletion should be expected to have a significant and potentially highly biased effect on the population of rRNA molecules remaining. All 3 rRNA species are transcribed in a single transcript that is then processed, so It is not surprising to detect 3’ ends that could be related to various processing intermediates. However, the prevalence of these intermediates could be highly skewed by the depletion step, so no conclusions should be drawn. This does not seem to be a major conclusion of the manuscript and should just be removed.

We thank the reviewer for raising this point. We agree that the ribo-depletion steps may have altered the appearance or rRNA 3’ ends, potentially enriching processing intermediates. We deemphasized this section in the revised manuscript and moved the results to supplemental data. The revised manuscript now more accurately states, “We detected a small amount of ribosomal rRNAs retained in the samples after rRNA depletion. Alignment of significant peaks assigned to rRNAs confirmed the ability of 3pMap to detect RNA 3’-ends accurately, and suggest alternative rRNA 3’ ends that may be used in precursor or mature rRNA (Supplementary Figure S2D-S2H)” (lines 265-2668).

2. The observation that uncharged tRNAs (free 3’-OH available for ligation to adapter) increase in the AZM-treated condition for several rare codon tRNAs is very interesting, but the relevant parameter for drawing connections to translation elongation efficiency and the stringent response is the ratio of charged to uncharged tRNAs so knowing only about the abundance of uncharged tRNA is not enough information to make these connections. It seems possible, for example, that rare codon tRNAs are simply upregulated under a condition of “translation stress” that might be caused by AZM. If this is the case, the abundance of charged tRNAs might also increase, thus increasing the overall concentration of charged rare codon tRNAs, which would have the opposite biological impact compared to the proposed interpretation. Either way, this result is interesting, but an additional experiment to assess the total abundance of those tRNAs or the ratio of charged to uncharged (by qPCR or northern blot) would significantly improve the ability to interpret the result.

We agree with the reviewer that we did not provide a balanced interpretation of this result in the initial manuscript. This point was also raised by reviewer #1 (point 4). While we considered additional experiments to characterize the ratio of charged to uncharged tRNA, we were unable to do such experiments due to decreased wet-lab capacity during the COVID-19 pandemic. To address this concern, we have substantially revised the manuscript to present both possible interpretations of increases in free tRNA 3’ ends.

3. The focus on “riboregulation”, which seems to generally refer to riboswitches and other examples of alternative RNA secondary structures playing regulatory roles, seems a bit misplaced. Computational prediction of RNA secondary structures without any experimental evidence to back those predictions up is not very reliable. Long stems in particular are suspect, given that they would require a huge length of nascent RNA to be exposed in order to form. As noted in the discussion, some of these predicted terminators could well be associated with termination of upstream genes, or even small RNAs, as was recently described in the leader of the RhlI gene. Small RNAs in general (outside a small handful of examples) are poorly annotated and little explored in Pseudomonas aeruginosa. Structures predicted from the genome sequence could also reflect functions of RNAs transcribed from the opposite strand. Concluding that any of the observed “early termination” events relate to the presence of novel riboswitches or alternative structures would require additional experiments, such as chemical probing of the RNA secondary structure or demonstration that mutations affecting the proposed structures affected the observed AZM phenotypes.

We agree that the original submission focused too heavily on riboregulators, considering that we did not have complementary experimental data to support regulatory roles. In the revised manuscript, we have moved the section on riboregulators to the end of the results to help refocus the manuscript on the transcriptional response to AZM. We agree that additional study would be needed to validate riboregulators. We believe the value of this section in our manuscript is in providing these putative elements, supported by defining their 3’ RNA ends, that could be the basis of future work. In the resubmission, we have also revised the language to reiterate that the predictions we make are putative, and further study is needed to evaluate their functions and examine alternative structure formation.

The reviewer’s point about 3’ ends formed from alternative transcription units is also important. To reduce false-positive riboregulator predictions from terminators associated with upstream genes, we restricted our search for early termination to transcript leaders that did not overlap upstream protein coding genes. Instead, we only searched for putative riboregulators in transcript leaders annotated by Wurtzel et al that did not overlap other genes. We agree that some early termination events could also reflect the production of small regulatory RNAs, like the sRNA produced from the leader of the rhlI gene and have added language describing this possibility to the discussion section (lines 563-564).

The general model that AZM-induced translational stalling leads to premature transcription termination in some transcripts, and that this mechanism relies on the formation of alternative secondary structures in the nascent transcripts is not unreasonable, but the data presented do not strongly support this model, and other alternatives must also be considered. For example, many of the computationally predicted alternative secondary structures coinciding with observed 3’ ends in 5’UTR regions are far upstream of the translation start, with no indication of uORFs. This makes it difficult to understand how translational stalling would be involved. The putative uORF shown for obgE requires a transcription start site substantially upstream of the annotated one, so its relevance is highly speculative.

We think the reviewer may have misunderstood our general model due to the organization of our initial manuscript. We did not mean to combine AZM-induced premature transcription termination in protein coding regions with alternative RNA structure formation in transcript leaders. In the revised manuscript, we have presented a more comprehensive discussion of potential models for the formation of premature 3’ ends in AZM-treated samples (Figure 4). We believe the reorganization and additional language including the models noted by the reviewers substantially improves the revised manuscript.

With respect to obgE, the reviewer raises an important issue about the location of the uORF. While it is not located in the annotated transcript leader, Our 3pMap data show RNA coverage extends beyond the annotated 5’ transcript leader, encompassing the putative uORF described in the manuscript new Figure S4. This may reflect slight differences in transcription start sites in our strain and the Wurtzel et al. annotations.

Other possibilities that are not addressed include:

1) cleavage of transcripts: the sequencing method used cannot distinguish between nascent transcript ends and cleavage events that leave a 3’-OH, and many bacterial ribonucleases have cleavage mechanisms that leave a 3’-OH. Many of the transcripts that were affected by AZM have also been described as targets of Hfq and/or Crc binding, and in some cases Hfq can recruit RnaseE to cleave the bound transcript. A connection between AZM treatment and Hfq/Crc mediated translational/RNA stability regulation would be very interesting, and seems at least as reasonable as a model to describe the data as the riboregulation model that is proposed.

We thank the reviewer for this suggestion and have added the possibility of nuclease activity in 3’ end formation in the revised manuscript (lines 496-507).

2) intrinsic pausing behavior of the RNA polymerase: while stabilized pausing events mediated by hairpin formation in the nascent RNA are described in the literature and contribute to rho-independent transcription termination, the RNA polymerase appears to pause very frequently without hairpin formation, and many transcriptional regulators can impact its behavior in these pauses. Interestingly, such pausing events have been proposed to be especially common at start codons, due to the biophysical characteristics of the DNA duplex over the sequence context of the shine-dalgarno sequence and the AUG start codon (Larson et al., Science 2014). It is interesting that several of the observed affected premature termination events occurred at start codons. (p)ppGpp/DksA and other secondary channel binding regulators have been proposed to affect behavior at transcription pause sites, so the possibility that ppGpp could affect the outcome of a pause at the obgE start codon is an intriguing one. Obviously, additional experiments would be needed to support such a model, but it seems at least as plausible as any of the other models presented in the manuscript.

We agree that the original submission did not adequately address transcriptional pausing and thank the reviewer for making this point and suggesting interesting alternative models. We have added language in the revised manuscript to raise the importance of transcriptional pausing, especially noting pausing at start codons (Larson et al) and the known influence of ppGpp on RNA polymerase pausing behavior (lines 546-547).

3) rho-dependent termination: in E. coli, rpoS expression has been suggested to be modulated by rho-dependent termination in the 5’UTR, which can be blocked by a small RNA. Obviously there are no data in the current study to support such a mechanism, but it is as possible as the proposed mechanisms.

We thank the reviewer for this suggestion. In the revised manuscript we have added language to describe the general possibility of sRNA regulation of termination, noting in the discussion that sRNAs antagonize rho-dependent termination in *E. coli rpoS* (lines 488-489).

A detailed mechanistic understanding of the regulatory events leading to changes in the abundances of different transcript 3’ ends may be outside the scope of this work, but the authors seem to have focused on a narrow category of mechanistic models to explain their data without any consideration of other models that are equally or even more plausible. The sentence in the discussion: “The candidate riboregulators we identified provide a first transcriptome-wide catalogue of potentially “druggable RNAs” in P. aeruginosa” in particular seems to be a dramatic overstatement of what is actually supported by the data.

We agree this was somewhat overstated and have substantially revised the language on riboregulators in the discussion to address this point.

The manuscript could be considerably strengthened by any of a number of follow-up experiments to support or refute proposed mechanisms: for example, the 5’ end of the obgE transcript could be mapped to determine whether a uORF is in fact present; the effect of the rhlR second codon mutation described in the discussion on the premature termination behavior reported here could be measured; the impact of a relA mutant on premature termination for a gene of interest could be assessed to determine the contribution of the stringent response. If the authors feel that performing these types of experiments to shore up their speculations about mechanism are outside the scope of the study, then their conclusions should be moderated and the discussion of possible models to explain their data should be broadened.

The reviewer makes good points about the interpretations we presented in the initial submission. For the revision, we have added qPCR validation of the shift in I/FL for hemN and *lasI* genes, and provide evidence that the putative uORF in obgE is included in RNA transcripts. We agree that our work raises a number of intriguing possibilities for future study, but do feel that followup work in those areas exceeds the scope of the study. As such, we have extensively revised the manuscript to moderate conclusions and expand our discussion of alternative models as suggested by the reviewer.

Minor Concerns:
1. In figures 2 and 4, there are small numbers just below the left end of the genome coordinates. The meaning of these numbers should be noted in the legend. I am guessing that they describe the range of the y-axis? If so, it may be a more fair representation of comparisons between different conditions to have the y-axes scaled the same. If this obscures some of the interesting features, then the different scaling is potentially important and should possibly also be noted in the text.

We apologize for any misunderstanding resulting from the IGV browser figure images. The figures are not intended to present absolute difference in expression. Rather, they show relative amounts of alternative 3’ ends. In the figures, browser images are presented using the “autoscale” feature of IGV. The numbers presented in the figure do indeed show the range of the y-axis, measured in raw readcount 3’ ends (not normalized by total reads per sample). We have added language to clarify this to the methods section of the revised manuscript (line 188).

2. Slowed penetration of biofilms has only been demonstrated for charged aminoglycoside antibiotics and not, to my knowledge, for azithromycin, so this is not a great justification for the increased treatment time of the biofilm cells. The authors do correctly note that the different treatment times could contribute significantly to the differences in observed effects, and it is not problematic to have two different treatments that survey a range of physiological states. However, I would recommend removing the statement about increased time required for penetration, because it perpetuates a belief that is not supported by data for azithromycin and that has been convincingly refuted for some antibiotics, including fluoroquinolones and tetracycline. Alternatively, a reference that includes biofilm penetration experiments with azithromycin should be included.

The reviewer makes an excellent point about this. We agree and have removed discussion of AZM penetration times from the revised manuscript.

**Have all data underlying the figures and results presented in the manuscript been provided?**
Large-scale datasets should be made available via a public repository as described in the *PLOS Genetics* [data availability policy](http://journals.plos.org/plosgenetics/s/data-availability), and numerical data that underlies graphs or summary statistics should be provided in spreadsheet form as supporting information.

Reviewer #1: Yes

Reviewer #2: Yes

PLOS authors have the option to publish the peer review history of their article ([what does this mean?](https://journals.plos.org/plosgenetics/s/editorial-and-peer-review-process#loc-peer-review-history)). If published, this will include your full peer review and any attached files.


If you choose “no”, your identity will remain anonymous but your review may still be made public.


**Do you want your identity to be public for this peer review?** For information about this choice, including consent to withdrawal, please see our [Privacy Policy](https://www.plos.org/privacy-policy).

Reviewer #1: No

Reviewer #2: No
