# Supplementary material for: Quantitative mapping of mRNA 3’ ends in Pseudomonas aeruginosa reveals a pervasive role for premature 3’ end formation in response to azithromycin
Source: PLoS Genet. 2021 Jul 12;17(7):e1009634. doi: 10.1371/journal.pgen.1009634 (PMC8297930; doi:10.1371/journal.pgen.1009634)
Supplement: Attachment — Submitted filename: Response_to_Reviewers_Revision2.docx [file pgen.1009634.s022.docx]

**Upload a Striking Image with a corresponding caption to accompany your manuscript if one is available (either a new image or an existing one from within your manuscript). If this image is judged to be suitable, it may be featured on our website. Images should ideally be high resolution, eye-catching, single panel square images.**

Reviewer's Responses to Questions

**Comments to the Authors:**

**Please note here if the review is uploaded as an attachment.**

Reviewer #1: In the revised manuscript entitled “Quantitative mapping of mRNA 3’ ends in Pseudomonas aeruginosa reveals a pervasive role for premature 3’ end formation in response to azithromycin” the authors present a much more focused and clear narrative and analysis of their data than exhibited by the original manuscript. The revised manuscript successfully focusses the narrative on response to azithromycin specifically. Most of my previous concerns were well addressed in the manuscript.

I only have one minor concern regarding the revised manuscript. However, as noted below, there are many problems with figure and supplementary figure mis-numbering (or just missing?) that make evaluation more challenging. I have noted some below, but the authors must go through the entire manuscript and check carefully.

We apologize for the mixup and have carefully checked all the figure numbers for the final revision.

My predominant minor concern is still the use of known riboswitches as a method for establishing a threshold for biologically relevant early termination. Yes, the threshold they use captures most riboswitches, but I question whether these regulators are expected to display significant early termination under the conditions examined given that most are not expected to act transcriptionally, and it is unclear what nutrient levels to expect in the rich medium conditions or what the predicted behavior of these regulators would be under such conditions (ON? OFF?, ?). Overall, this is a relatively minor concern in the context of the entire work, but I still believe this is not a robust strategy for establishing such a threshold.

The reviewer makes a very good point. Indeed, it is difficult to predict whether any specific riboswitches and attenuators are expected to be active in the planktonic or biofilm conditions in our study. Even though we used defined media when inoculating the cultures, by the time the cells are harvested, the media composition may have changed due to cellular metabolism. Furthermore, even for riboswitches that have been well-characterized in other species, it is difficult to predict what concentrations of metabolites may be required for their activity in P. aeruginosa. We have now revised the results section to reiterate that our cutoff is heuristic in nature (line 415). We also noted in the discussion (line 590) that our heuristic cutoff could miss elements that aren’t active under our culture conditions. We believe this will help readers appreciate the reviewers point.

Figure mis-numberings:

Line 300: “Figure 3C” I think should refer to Figure 2C? – Corrected

Line 323: “Figure 3E” I think should refer to Figure 2E? - Corrected

Line 409: The reference to “Supplementary Figure S3B” is incorrect. Corrected.

Line 413: The reference to “Supplementary Figure S4A” is incorrect. Corrected

Line 420: Reference to “Supplementary Figure S4D-F” is not correct. Corrected

Table 1 is not included in the manuscript. We thank the reviewer for pointing out this issue, which is rectified in the revised manuscript. We moved the original Table 1 to Table S13.

The review package only included Supplementary Tables 1-7 and Supplementary Figures 1-5. Therefore, the following calls are to missing tables/figures (and there may be additional references that I did not catch).

We apologize for the mix-up, which may have occurred during the file upload process. The full set of revised supplementary tables should now be available.

Line 247/248: Supplementary Figure 11A – changed the reference to Supplementary Figure 5A, Supplementary Table 11 (The revised supplementary file has this table)

Line 413:Supplementary Table 11 (The revised supplementary file has this table)

Line 412: Supplementary Table 12 (The revised supplementary file has this table)

Other areas in need of clarification:

Line 205: “…n=400 for mRNA, n=100 for tRNAs and mRNAs as the intergenic spaces….”, unclear which mRNAs use 400 vs 100. I suspect this may be a typo. This was a typo and is corrected in the revised manuscript.

Line 269: The manuscript references three classes of genes, but I only see two (tRNA and protein coding sequences) actually mentioned. Is there a third that is missing?

Three categories included tRNAs, rRNAs, and mRNAs. The manuscript now contains the revised version.

Line 81: A comma after pauses in the following sentence would increase clarity: “transcription termination, transcriptional pauses and stable intermediates resulting from ribonucleases”

We thank the reviewer for the suggestion and have edited the manuscript to incorporate the change.

Line 412: typo: Predicted by Riboswitch scanner. Corrected the typo

Reviewer #2: I would like to thank the authors for their substantial revisions to the manuscript and supplemental tables. I agree that it is greatly improved and I really enjoyed reading this new version. The dataset is of great value for many researchers and opens exciting avenues for further exploration. I note below some extremely minor typographical errors that the authors may wish to correct. Also, perhaps through a file management error, the supplemental tables that were available to me were the originally submitted ones rather than the substantially revised ones that were extensively described in the authors' response to reviewers. I believe that the authors created the new supplements and I very much look forward to exploring them. The authors and the journal should ensure that these new tables are included with the final published article.

Very minor errors:

Line 131: Probably should be 20-40 micrograms of total RNA

We thank the reviewer and have now corrected this in the revised manuscript.

Line 269: “three classes of genes” but then only 2 listed. Later in the paragraph rRNA is mentioned in addition to the tRNA and protein coding sequences. This should just be made consistent.

The manuscript now contains the revised version that reads “We assessed the distribution of 3’ significant peaks in intragenic vs. intergenic regions of tRNAs and protein coding sequences (Supplementary Figure S2)”.

Line 412: awkward wording/typo Corrected

Line 581: typo Corrected

**Have all data underlying the figures and results presented in the manuscript been provided?**

Large-scale datasets should be made available via a public repository as described in the *PLOS Genetics* [data availability policy](http://journals.plos.org/plosgenetics/s/data-availability), and numerical data that underlies graphs or summary statistics should be provided in spreadsheet form as supporting information.

Reviewer #1: Yes

Reviewer #2: Yes

PLOS authors have the option to publish the peer review history of their article ([what does this mean?](https://journals.plos.org/plosgenetics/s/editorial-and-peer-review-process#loc-peer-review-history)). If published, this will include your full peer review and any attached files.

If you choose “no”, your identity will remain anonymous but your review may still be made public.

**Do you want your identity to be public for this peer review?** For information about this choice, including consent withdrawal, please see our [Privacy Policy](https://www.plos.org/privacy-policy).

Reviewer #1: No

Reviewer #2: No
